# Guillain-Barré Syndrome in Suriname; Clinical Presentation and Identification of Preceding Infections

**DOI:** 10.3389/fneur.2021.635753

**Published:** 2021-02-10

**Authors:** Thomas Langerak, Irene van Rooij, Laura Doornekamp, Felicity Chandler, Mark Baptista, Harvey Yang, Marion P. G. Koopmans, Corine H. GeurtsvanKessel, Bart C. Jacobs, Barry Rockx, Kirsten Adriani, Eric C. M. van Gorp

**Affiliations:** ^1^Department of Viroscience, Erasmus Medical Center, Rotterdam, Netherlands; ^2^Department of Neurology, St. Vincentius Hospital, Paramaribo, Suriname; ^3^Department of Neurology, Academic Hospital Paramaribo, Paramaribo, Suriname; ^4^Department of Neurology, Erasmus Medical Center, Rotterdam, Netherlands; ^5^Department of Immunology, Erasmus Medical Center, Rotterdam, Netherlands; ^6^Department of Neurology, Zaans Medisch Centrum, Zaandam, Netherlands

**Keywords:** Guillain-Barré syndrome, Suriname, Zika virus, dengue virus, arthropod borne viruses

## Abstract

Guillain-Barré syndrome (GBS) is associated with various types of preceding infections including *Campylobacter jejuni* and cytomegalovirus, but there is also an association with arthropod borne viruses (arboviruses), such as Zika virus, that are endemic in tropical regions. Here we present the clinical characteristics of 12 GBS patients from Suriname that were hospitalized between the beginning of 2016 and half 2018. Extensive diagnostic testing was performed for pathogens that are commonly associated with GBS, but also for arboviruses, in order to identify the preceding infection that might have led to GBS. With this extensive testing algorithm, we could identify a recent infection in six patients of which four of them had evidence of a recent Zika virus or dengue virus infection. These results suggest that arboviruses, specifically Zika virus but possibly also dengue virus, might be important causative agents of GBS in Suriname. Furthermore, we found that more accessibility of intravenous immunoglobulins or plasma exchange could improve the treatment of GBS in Suriname.

## Introduction

Guillain-Barré syndrome (GBS) is an immune-mediated polyradiculoneuropathy, characterized by a rapidly progressive symmetrical limb weakness and decreased or absent deep tendon reflexes (1). GBS can be a life-threatening disease because of respiratory and autonomic failure, and has an estimated mortality of 3–7% (2). The exact pathogenesis of GBS is unknown, but it is thought that preceding infections or vaccinations may trigger the production of autoantibodies to components of peripheral nerves due to molecular mimicry, leading to peripheral nerve injury (1). There are multiple clinical variants and electrophysiological GBS subtypes, such as acute inflammatory demyelinating polyneuropathy (AIDP), acute motor axonal neuropathy (AMAN), acute motor and sensory axonal neuropathy (AMSAN) and Miller Fisher syndrome (1). Diagnosis of GBS is based on clinical characteristics but can be supported by investigation of cerebrospinal fluid (CSF) and nerve conduction studies (3). Diagnosis and classification of GBS can be challenging because of the heterogeneity of the syndrome and the extensive differential diagnosis. Proven effective treatment of GBS are intravenous immunoglobulins and plasma exchange (1, 3).

Multiple pathogens are associated with GBS such as *Campylobacter jejuni* (*C. jejuni*), Epstein-Barr virus (EBV), cytomegalovirus (CMV), *Mycoplasma pneumoniae* (*M. pneumoniae*) and hepatitis E virus (HEV) (4). The type of preceding infection is related to the clinical presentation and course of GBS, and the variety of preceding infections contributes to the diversity in clinical variants and prognosis. Besides the above mentioned pathogens that are associated with GBS, during the 2015–2016 Zika virus (ZIKV) outbreak in the Americas, it became clear that this virus is also associated with GBS (5, 6). More recently, an association between the newly emerged severe acute respiratory syndrome coronavirus 2 (SARS-CoV-2), and GBS was suggested (7, 8).

Diagnosis of the preceding infection that might have triggered GBS can be difficult. Firstly, direct pathogen detection, for example, with polymerase chain reaction (PCR), is quite often not possible when GBS is diagnosed because of the time lag of 1–4 weeks between the initial infection and onset of GBS symptoms. Furthermore, diagnosis of preceding infections of GBS with serology can be challenging due to cross-reactivity of antibodies between certain pathogens and reactivation of non-related antibodies during infection.

Here we describe 12 patients from Suriname with GBS who were recruited in a prospective observational study taking place from February 2016 to June 2018. Suriname is a country in South-America which has, due to the tropical climate, a relative high burden of arthropod borne viruses (arboviruses) like ZIKV and dengue virus (DENV) (9, 10). The aim of this study was to describe the clinical presentation, treatment and outcomes of GBS patients in Suriname and to diagnose the preceding infections that lead to GBS in these patients. We were specifically interested in the role of arboviruses as potential preceding infections that can lead to GBS. The results of this study may result in more knowledge about the clinical presentation and the etiology of preceding infections of GBS in Suriname which could help to improve diagnostic preparedness and treatment of GBS in Suriname, and possibly other countries in South America.

## Materials and Methods

### Participants

Patients with suspected GBS were recruited for this prospective study as soon as possible after clinical GBS suspicion. Participants were recruited in all three hospitals in Paramaribo, Suriname, from February 2016 until July 2018. The case definitions of the Brighton Collaboration were used to determine the level of diagnostic certainty of GBS ranging from 1 (most certainty for GBS diagnosis) to 4 (least certainty for GBS diagnosis) (2). If during admission another diagnosis than GBS was made, or of if insufficient clinical information was available to verify the GBS diagnosis, these participants were excluded, as were participants from whom no blood was collected. One participant was initially suspected of a paraparetic form of GBS with paralysis of the legs and normal strength of the arms. This diagnosis was changed during admission to transverse myelitis because of a sensory level at Th5 and CSF pleiocytosis of 206 cell/μl and no further suspicion of GBS. Even though concomitant GBS and transverse myelitis can occur, this patient did not meet the Brighton diagnostic criteria for GBS and was therefore excluded from this study (11–13). Three patients (patient 1, 2, and 6) were previously described in a smaller case series on possible ZIKV associated GBS in Suriname (14).

### Data and Sample Collection

Serum and plasma were collected at enrolment in this study and 10–14 days later, or at the day of hospital discharge if this was before day 10. Neurological examination was performed by one of the researchers every seven days until hospital discharge. Additional information regarding medical history, (onset of) symptoms, and antecedent events like infections or vaccinations was collected with a questionnaire. Data from nerve conduction studies and analysis of cerebrospinal fluid were, when available, collected from all participants.

### Ethics

This study protocol was approved by the ethical board of the Ministry of Health in Suriname. Informed consent was obtained from all patients and in case the patient was younger than 16 years old, from their parents or representatives. The study was carried out in accordance with the Declaration of Helsinki.

### Diagnostic Tests

Serum samples were tested for presence of antibodies against pathogens commonly associated with GBS; *C. jejuni*, EBV, CMV, HEV and *M. pneumoniae*. Furthermore, serological tests were performed to diagnose (recent) infections with the arboviruses ZIKV, DENV and chikungunya (CHIKV). All the serological tests -except serology for *C. jejuni*- were performed at the department of Viroscience at Erasmus MC, Rotterdam, the Netherlands.

Presence of IgM antibodies against ZIKV and DENV and IgM and IgG antibodies against HEV and *M. pneumoniae* were assessed with use of commercial ELISA kits (ZIKV and DENV; Euroimmun, HEV; Wantai Biological, *M. pneumoniae*; Serion Diagnostics). EBV and CMV serology was performed using a chemiluminescent immunoassay (DiaSorin LIAISON®). Chikungunya antibodies were detected with an indirect immunofluorescence test (Euroimmun). All these assays were performed according to the manufacturer's instructions. In case of a positive IgM response for EBV or CMV, additional testing, detection of Epstein-Barr virus Nuclear Antigen (EBNA) antibodies and a CMV avidity test respectively, was performed to determine if this was likely to be a primary infection or not. *C. jejuni* serology was performed with an indirect IgG ELISA and antibody class capture ELISAs for IgM and IgA antibodies at the Department of Medical Microbiology, Reinier de Graaf Gasthuis, Delft, the Netherlands.

Neutralizing antibodies against ZIKV and DENV-2 [used as a representation of total DENV immunity (15)], were determined with an in-house micro-neutralization test (VNT) as previously described (9). Sera were tested in triplicates and the geometric mean of the highest final serum dilution was reported as titer. For both ZIKV and DENV-2, the cut-off of a positive VNT was a final serum dilution >1:32.

For ZIKV diagnosis, a reverse transcriptase polymerase chain reaction (RT-PCR) was performed on plasma or, when available, urine using the primer/probe set described by Lanciotti et al. (16).

### Case Definitions for Preceding Infections

For the interpretation of the serological and molecular tests performed in these patients, a distinction was made between confirmed recent infections, probable recent infections and possible recent infections. A recent infection was considered confirmed if one serum or urine sample was RT-PCR positive, or if IgM antibodies against the specific pathogens were present and there was a more than four-fold increase of IgG- or neutralization titer in paired samples. Presence of IgM antibodies with an increasing IgG- or neutralization titer in paired samples was considered a probable recent infection. Presence of IgM but with no increase in IgG or neutralization titer in paired samples was considered a possible recent infection. When paired blood samples were not available, presence of IgM in a single blood sample was considered a possible recent infection. Specifically for CMV, presence of anti-CMV IgM in combination with a rising IgG titer with low avidity was considered a confirmed recent CMV infection. The combination of CMV IgM with IgG with high avidity was considered as reactivation of CMV antibodies and not a recent infection (17). Presence of IgM against EBV in combination with IgG against EBV nuclear antigen (EBNA) was considered a non-recent infection (18).

## Results

### Participant Enrolment

**Figure 1:**
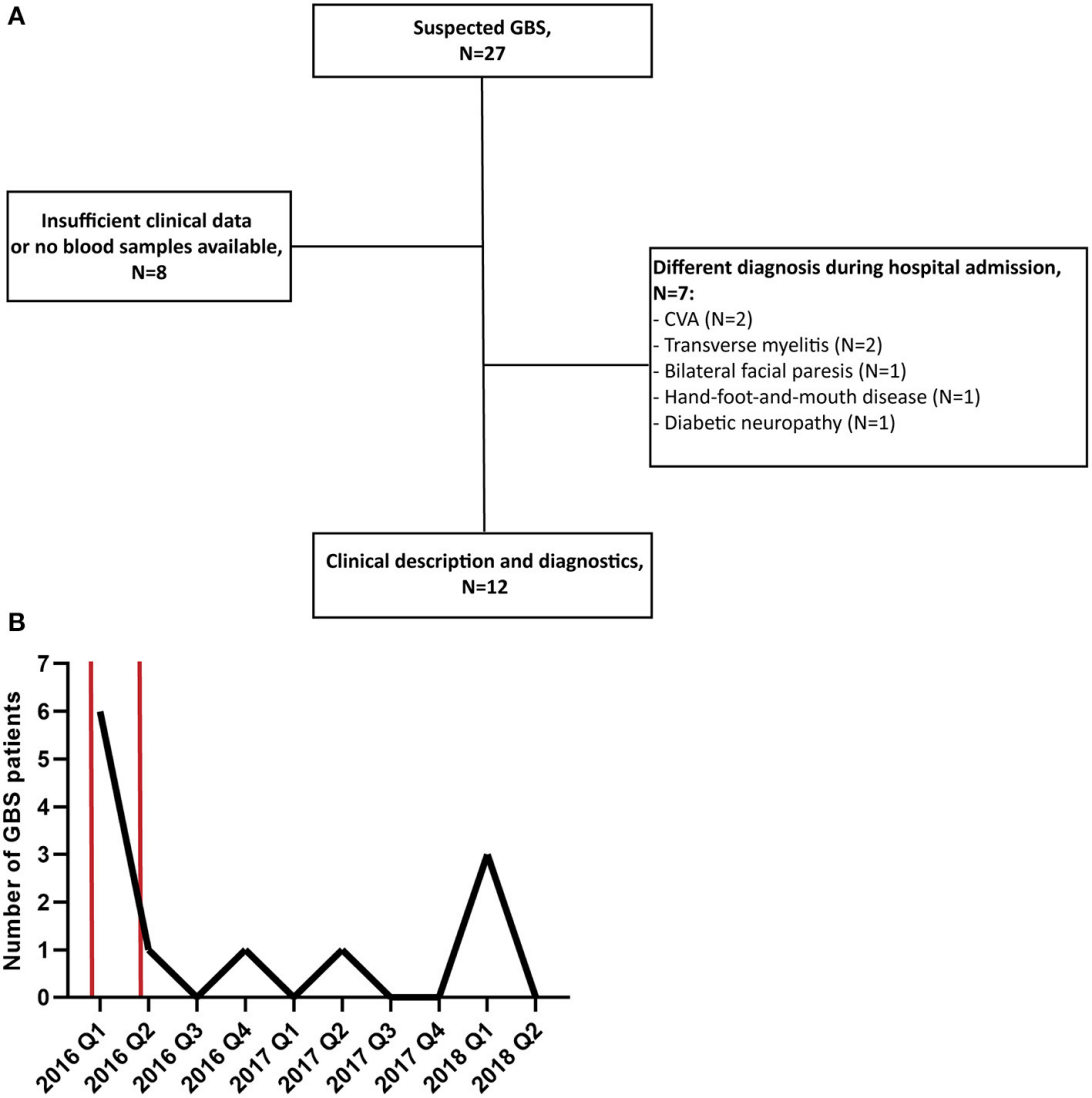
**(A)** Flowchart of patient recruitment and exclusion. **(B)** Number of GBS patients recruited in this study per quarter of a year (Q). The time period of the peak of the ZIKV outbreak in Suriname is marked with two red bars. CVA, cerebrovascular accident.

In total, 27 patients with suspected GBS were enrolled in this study. Of these 27 patients, 15 were excluded because during admission another diagnosis than GBS was made or because the availability of clinical data or blood samples was insufficient, illustrated by the flowchart in Figure 1A. The number of GBS patients recruited per quarter of a year is displayed in Figure 1B. The highest peak of GBS patients, in the first quarter of 2016, coincided with the peak of the ZIKV outbreak in Suriname and South America in the first months of 2016 (19, 20).

### Clinical Characteristics

Seven out of 12 patients (58.3%) had a pure motor form of GBS (without sensory deficits, Table 1). Cranial nerves were affected in five patients (41.7%), and a facial palsy was present in four of these patients (80.0%). At nadir, six patients (50.0%) were confined to bed or chair of which three patients (50.0%) required assisted mechanical ventilation. Five patients (41.7%) received treatment with intravenous immunoglobulins (IVIg) while the other seven patients (58.3%) only received supportive care. None of the patients were treated with plasma exchange. Eight out of 12 patients (66.7%) underwent nerve conduction studies and electromyography during admission of which five (62.5%) showed a demyelinating type of GBS, one (12.5%) showed an axonal GBS type, one (12.5%) was classified as equivocal and in one (12.5%) the potentials were absent. Brighton criteria 1 (highest level of diagnostic certainty) for GBS diagnosis was met in six patients (50.0%), level 2 in five patients (41.7%) and level 3 in one patient (8.3%). Median hospital stay was 14 days (IQR 11–34 days).

**Table 1:** Clinical characteristics of GBS patients.

**Patient**	**1**	**2**	**3**	**4**	**5**	**6**
**A**						
Sex, Age (yr)	M, 46	M, 44	F, 67	F, 54	M, 12	M, 64
Start of GBS symptoms	09-Feb-2016	10-Feb-2016	19-Feb-2016	09-Feb-2016	22-Feb-2016	30-Jan-2016
Antecedent symptoms	Fever, rash, arthralgia, conjunctivitis	Influenza-like symptoms, double vision	Fever, myalgia	None	Influenza-like symptoms	Myalgia and arthralgia
Clinical subtype	PM	SM	PM	PM	SM	SM
Cranial nerve involvement	Facial nerve, glossopharyngeal nerve	No	Facial nerve	No	No	Facial nerve
CSF cell count	0/μl	33/μl	3/μl	56/μl	0/μl	16/μl
Protein in CSF	1.98 g/L	4.4 g/L	0.5 g/L	2.33 g/L	0.18 g/L	2.93 g/L
NCS result	Demyelinating	Demyelinating	-	-	-	Equivocal
GBS disability score^1^ at nadir	2	3	4	4	3	4
Diagnostic certainty GBS diagnosis (2)	1	1	2	3	2	1
IVIg/PE treatment	No	No	No	No	No	No
Preceding infection	Unknown	Confirmed ZIKV	Probable ZIKV, possible DENV	Unknown	Confirmed DENV	Unknown
**Patient**	**7**	**8**	**9**	**10**	**11**	**12**
**B**						
Sex, Age (yr)	F, 39	F, 20	F, 10	M, 48	M, 58	F, 58
Start of GBS-symptoms	19-Apr-2016	14-Nov-2016	23-May-2017	14-Jan-2018	23-Jan-2018	24-Feb-2018
Antecedent symptoms	None	Fever, diarrhea, vomiting	Conjunctivitis	Influenza-like symptoms	Diarrhea, vomiting	Fever, myalgia
Clinical subtype	SM	PM	PM	SM	PM	PM
Cranial nerve involvement	No	No	Oculomotor nerve, facial nerve	No	No	No
Cells in CSF	0/μl	4/μl	2/μl	3/μl	2/μl	3/μl
Protein in CSF	2.09 g/L	2.56 g/L	0.16 g/L	0.30 g/L	0.90 g/L	0.66 g/L
NCS result	Demyelinating	Demyelinating	-	Axonal	Absent potentials	Demyelinating
GBS disability score^1^ at nadir	4	5	5	4	5	4
Diagnostic certainty GBS diagnosis (2)	1	1	2	2	2	1
IVIg / PE treatment	Yes, IVIg	Yes, IVIg	Yes, IVIg	Yes, IVIg	Yes, IVIg	No
Preceding infection	Unknown	Confirmed CMV, possible ZIKV/DENV	Probable *M. pneumoniae*, possible *C. jejuni*	Unknown	Possible *C. jejuni*/*M. pneumoniae*.	Unknown

*A, patient 1–6; B, patient 7–12*.

*M, male; F, female; PM, pure motor; SM, sensory-motor; -, not performed; NCS, nerve conduction studies; IVIg, intravenous immunoglobulins; PE, plasma exchange*.

1 *GBS disability score. 0, healthy; 1, minor symptoms or signs of neuropathy but capable of manual work/capable of running; 2, able to walk without support of a stick but incapable of manual work/running; 3, able to walk with a stick, appliance or support; 4, confined to bed or chair bound; 5, requiring assisted ventilation (for any part of the day or night); 6, death*.

### Preceding Infections

Nine patients (75%) reported symptoms of a possible preceding infection and the average time between these symptoms and the first neurological symptoms was 9 days (range 5–22 days). Most of the patients (*N* = 5, 41.6%) reported non-specific, flu-like symptoms such as fever and myalgia. Two patients (16.7%) reported symptoms of a gastrointestinal infection while two other patients reported symptoms associated with an arbovirus infection, such as conjunctivitis and skin rash. The interpretation of the diagnostic test results are shown in Table 1 and the results of the diagnostic tests are shown in Table 2. In three patients (25%) we could confirm the preceding infection based on the criteria described in the methods. Of these three, patient 2 had a confirmed recent infection with ZIKV, patient 5 had a confirmed recent infection with DENV and patient 8 had a confirmed recent infection with CMV. Two patients (16.7%) had a probable recent infection, one with *M. pneumoniae* (patient 9) and one with ZIKV (patient 3) although a recent DENV infection could not be ruled out in this last patient. In one patient (8.3%, patient 11) we found evidence of a possible recent infection with *M. pneumoniae* and/or *C. jejuni*. Finally, in six patients (50.0%) we could not find sufficient evidence of a recent infection based on our diagnostic criteria. Four of these six patients (patient 1, 6, 10, and 12) did have non-specific symptoms of an infection, such as fever and myalgia, prior to the start of GBS symptoms. None of the patients reported to have recently received a vaccination.

Table 2Results of diagnostic tests for GBS preceding infections.

**Table:** 

**Patient**		**Days since onset of preceding symptoms**	***Campylobacter jejuni***			**Cytomegalovirus**			**Epstein-Barr virus**			**Hepatitis E virus**		***Mycoplasma pneumoniae***	
			**IgM**	**IgA**	**IgG**	**IgM**	**IgG**	**Avidity**	**IgM**	**IgG VCA**	**IgG EBNA**	**IgM**	**IgG**	**IgM**	**IgG**
**A**															
**1**		15	*N*	*N*	*P* (1.5)	*N*	*P* (92.5)	-	*N*	*P* (>750)		-	-	*N*	*P* (21.6)
**2**	**1**^**st**^	24	*N*	*N*	*P* (0.7)	*N*	*P* (93.3)	-	*N*	*P* (249)		*N*	*N*	-	-
	**2**^**nd**^	26	-	-	-	-	-	-	-	-		-	-	-	-
**3**	**1**^**st**^	11	*N*	*N*	*P* (0.9)	*P* (31.0)	*P* (79.1)	High (0.64)	*P* (50.0)	*P* (505)	*P* (68.0)	*N*	*N*	*N*	*P* (15.1)
	**2**^**nd**^	17	*N*	*N*	*P* (0.8)	*P* (29.9)	*P* (69.5)	-	*P* (73.9)	*P* (>750)	*P* (45.4)	*N*	*N*	*N*	B (10.6)
**4**	**1**^**st**^	-	*N*	*N*	*P* (0.5)	*N*	*P* (158)	-	*N*	*P* (22.6)		*N*	*N*	*N*	*P* (19.6)
	**2**^**nd**^	- (12 days between 1^st^ and 2^nd^ sample)	*N*	*N*	*P* (0.4)	*N*	*P* (173)	-	*N*	*N*		*N*	*N*	*N*	B (12.3)
**5**	**1**^**st**^	11	*N*	*N*	*P* (1.2)	*N*	*P* (103)	-	*N*	*P* (203)		*N*	*P*	*N*	B (12.2)
	**2**^**nd**^	20	*N*	*N*	*P* (0.7)	-	-	-	-	-		-	-	*P* (49.1)	*N*
**6**	**1**^**st**^	39	*N*	*N*	*P* (1.7)	*N*	*P* (90.5)	-	*N*	*P* (>750)		*N*	*N*	*N*	*P* (15.9)
	**2**^**nd**^	53	*N*	*N*	*P* (1.2)	*N*	*P* (118)	-	*N*	*P* (710)		*N*	*N*	*N*	*P* (27.0)
**7**	**1**^**st**^	-	*N*	*N*	*P* (1.8)	*N*	*P* (>180)	-	*N*	*P* (>750)		*N*	*P* (2.1)	*N*	*P* (24.6)
	**2**^**nd**^	−10 days between 1^st^ and	-	-	-	*N*	*P* (>180)	-	*N*	*P* (>750)		-	-	*N*	*P* (33.8)
**8**	**1**^**st**^	20	*N*	*N*	*P* (2.4)	P(123)	*P* (69)	Low(0.09)	B (36.2)	*P* (314)		*N*	*N*	B (13.4)	*P* (19.7)
	**2**^**nd**^	32	*N*	*N*	*P* (2.2)	P(85.8)	*P* (115)	-	*N*	*P* (313)		*N*	*N*	*N*	B (12.1)
**9**	**1**^**st**^	7	P(0.25)	*P* (1.9)	P(2.7)	*N*	*P* (80.5)	High (0.77)	*N*	*P* (218)		*N*	*N*	P(46.6)	B(14.5)
	**2**^**nd**^	17	*N*	*P* (.25)	P(2.6)	*P* (37.8)	*P* (112)	-	*N*	*P* (455)		*N*	*P* (5.6)	P(51.6)	P(38.2)
**10**		31	*N*	*N*	*P* (1.7)	*N*	*P* (126)	-	*N*	*P* (>750)		*N*	*P* (1.4)	*N*	*P* (41.0)
**11**	**1**^**st**^	9	P(0.44)	*P* (2.4)	P(2.6)	*N*	*P* (177)	-	*N*	*P* (>750)		*N*	*P* (1.1)	-	P(42.5)
	**2**^**nd**^	20	*N*	*P* (.81)	P(2.6)	*N*	*P* (157)	-	*N*	*P* (>750)		*N*	*P* (1.8)	P(40.3)	P(37.6)
**12**		17	*N*	*N*	*P* (2.3)	*N*	*P* (148)	-	*N*	*P* (>750)		*N*	*P* (9.5)	*N*	*P* (51.6)

**Table:** 

**Patient**		**Days since onset of preceding symptoms**	**Zika virus**				**Dengue virus**		**Chikungunya virus**	
			**IgM**	**VNT**	**PCR plasma**	**PCR urine**	**IgM**	**VNT**	**IgM**	**IgG**
**B**										
**1**		15	*N*	*P* (1:161)	*N*	*N*	*N*	*P* (1:64)	*N*	*N*
**2**	**1**^**st**^	24	*N*	*P* (1:80)	-	P	*N*	*P* (1:162)	*N*	*P* (>100)
	**2**^**nd**^	26	*N*	*P* (1:102)	-	-	*N*	*P* (1:204)	*N*	*P* (>100)
**3**	**1**^**st**^	11	P(5.4)	P(1:101)	*N*	*N*	P(12.2)	P(1:812)	*N*	*P* (10)
	**2**^**nd**^	17	P(2.8)	P(1:203)			B(0.9)	P(1:646)	*N*	*P* (10)
**4**	**1**^**st**^	-	*N*	*N*	*N*	-	*N*	*N*	*N*	*N*
	**2**^**nd**^	- (12 days between 1^st^ and 2^nd^ sample)	*N*	*N*	-	-	*N*	*N*	*N*	*N*
**5**	**1**^**st**^	11	*N*	*N*	*N*	*N*	*N*	P(1:64)	*N*	*P* (>100)
	**2**^**nd**^	20	*N*	*N*			P(1.33)	P(1:408)	*N*	*P* (>100)
**6**	**1**^**st**^	39	*N*	*P* (1:323)	-	*N*	*N*	*P* (1:812)	*N*	*P* (>100)
	**2**^**nd**^	53	*N*	*P* (1:323)	-	-	*N*	*P* (1:646)	*N*	*P* (>100)
**7**	**1**^**st**^	-	*N*	*N*	*N*	-	*N*	*P* (1:64)	*N*	*N*
	**2**^**nd**^	- (10 days between 1^st^ and 2^nd^ sample)	*N*	*N*			*N*	*P* (1:102)	*N*	*N*
**8**	**1**^**st**^	20	P(2.4)	N	*N*	-	P(2.35)	P(1:162)	*N*	*N*
	**2**^**nd**^	32	B(1.0)	N	-	-	P(1.25)	P(1:102)	*N*	*N*
**9**	**1**^**st**^	7	*N*	*N*	*N*	-	*N*	*P* (1:40)	*N*	*P* (>100)
	**2**^**nd**^	17	*N*	*N*	-	-	*N*	*P* (1:80)	*N*	*P* (>100)
**10**		31	*N*	*N*	-	-	*N*	-	*N*	*P* (10)
**11**	**1**^**st**^	9	*N*	*P* (1:203)	*N*	-	-	-	-	-
	**2**^**nd**^	20	*N*	*P* (1:128)	-	-	*N*	-	*N*	*N*
**12**		17	*N*	*N*	*N*	-	*N*	*P* (1:162)	*N*	*N*

*A, diagnostic results of pathogens commonly associated with GBS; B, diagnostic results of arthropod borne viruses that are possibly associated with GBS. Results on which the diagnosis was based are marked bold and in red. 1^st^, first sample; 2^nd^, second sample; VCA, viral capsid antigen; EBNA, Epstein Barr nuclear antigen; VNT, virus neutralization test; P, positive; N, negative; B, Borderline; -, not performed*.

## Discussion

### Diagnosis and Clinical Presentation GBS

In this study, we recruited patients as soon as the treating neurologist suspected GBS. In a relatively large amount of the patients, another diagnosis than GBS was subsequently made by the treating physician and these patients were excluded from this study. This demonstrates that diagnosing GBS, especially in low- and middle income countries with sometimes limited diagnostic facilities, can be challenging and that other conditions need to be ruled out before the diagnosis of GBS can be made. Because of the limited sample size of this study it is difficult to draw conclusions from the clinical data of these patients. In general, the diversity in clinical presentation of the described patients corresponds with what is known about GBS (1). One observation that can be made is that only five GBS patients were treated with IVIg while 11 patients were unable to walk or wheel chair bound and had an indication for treatment (1, 3). This undertreatment is explained by the limited availability of IVIg or plasma exchange facilities in Suriname.

### Preceding Infections of GBS

We performed extensive diagnostic testing for preceding infections that are commonly associated with GBS and arboviruses that are endemic in Suriname and are possibly associated with GBS. From 9 out of 12 patients we could collect paired serum samples during hospital admission. As a result, we found indications of a preceding infection in 6 of the 12 GBS patients (50.0%). Four of these six patients (66.7%) in which we could diagnose a recent infection had evidence of a recent ZIKV or DENV infection. It is possible that this relatively high percentage of possible ZIKV associated GBS cases is because recruitment of patients for this study started during the peak of the ZIKV outbreak in Suriname at the beginning of 2016. As is indicated in Figure 1A, during this period there was a peak in GBS patients that were recruited for this study. From the results of the serological tests in Table 2, it can be concluded that it is difficult to distinguish a recent ZIKV infected from a recent DENV infection based on serology because of cross-reactivity of anti-flavivirus antibodies. In this study we used neutralization assays for DENV-2 and ZIKV serology which is the gold standard for flavivirus serology and has shown to give less cross-reactivity than, for example, ELISA tests (9, 15, 21). Another diagnostic challenge is that asymptomatic reactivation of CMV and EBV can occur during acute infections with other pathogens which can lead to the presence of anti-CMV or EBV IgM antibodies (17, 22). In order to differentiate between a primary CMV or EBV infection or reactivation of these viruses, it is possible to measure the avidity of IgG antibodies against CMV, which are low after a recent infection, and to test for presence EBNA IgG antibodies against EBV which are not present after recent EBV infection (17, 18).

Interestingly, in patient 5 we found IgM antibodies against DENV and a more than six-fold increase in the neutralizing antibody titer in paired serum samples taken nine days apart from each other. No IgM or neutralizing antibodies against ZIKV were detected. Based on these results and the fact that this 12 year old patient did not recently receive a yellow fever vaccination, he was classified as having a confirmed recent DENV infection. The patient presented with pain in the backside of both legs, bilateral foot drop and difficulty walking. During physical examination, symmetrical areflexia and pain in the lower extremities was found, the Lasègue sign was positive and no neck stiffness was observed. The CSF did not contain white blood cells or elevated albumin levels and the CSF culture was negative. An EMG was not performed in this patient. DENV has previously sporadically been associated with GBS and other neurological complications (23–25). It might be worthwhile to study this possible association in more detail with specific attention to the clinical presentation of suspected GBS or a GBS-like syndrome caused by DENV.

Besides ZIKV and DENV, we also found evidence of preceding infections with pathogens that are commonly associated with GBS; CMV, *M. pneumoniae* and *C. jejuni*. In two patients (9 and 11) we found evidence of a possible recent infection with both *M. pneumoniae* and *C. jejuni*. For *C. jejuni*, IgM was only positive in the first collected sample from both patients, this can indicate that the possible *C. jejuni* infection was not very recent. However, it has been shown that IgM antibodies against *C. jejuni* can be short lived or even absent, especially in asymptomatic infections (26). A recent *M. pneumoniae* infection was probable in patient 9 because of the presence of anti *M. pneumoniae* IgM antibodies and a rise in the IgG titer. In patient 11, a recent infection with *M. pneumoniae* was less likely since, even though IgM antibodies were present in the first sample, no kinetics were observed in the anti *M. pneumoniae* IgG titers. We did not find evidence of recent infections with HEV, EBV or CHIKV in any of these patients. In patient 1 and 6 we could not make a diagnosis of a preceding infection based on the diagnostic criteria described above. However, both patients already had a high titer of neutralizing antibodies against ZIKV early in the ZIKV outbreak (20). This could be indicative of a recent ZIKV infection since ZIKV did not circulate in Suriname before the end of 2015 and both patients reported to have had symptoms that are associated with arboviral infection such as myalgia and arthralgia. However, since it has been shown that after a recent DENV infection, cross-neutralization of ZIKV can occur in some cases, it is also possible that these two patients had a recent DENV infection (15, 21).

### Strengths and Limitations

A strength of this study is that we performed extensive and state of the art, diagnostic testing to try to identify preceding infection that might have triggered GBS. Because of this extensive testing we were able to demonstrate the challenges that arise with the interpretation of serological diagnostic results. A limitation is that the small sample size of this study and the period of recruitment of the patients (partially during the ZIKV outbreak) does not allow us to generalize the results found in this study with respect to amongst others the incidence of GBS in Suriname and the exact contribution of the different pathogens in causing GBS. Furthermore, because of insufficient clinical data, we had to exclude a relatively large amount of study participants.

## Conclusion

In conclusion, we found that—apart from infections with pathogens that are commonly associated with GBS—infections with ZIKV and possibly DENV might play an important role in causing GBS in Suriname. Furthermore, more accessibility to IVIg or plasma exchange could improve the treatment of GBS in Suriname.

## Data Availability Statement

The original contributions presented in the study are included in the article/supplementary material, further inquiries can be directed to the corresponding author/s.

## Ethics Statement

The studies involving human participants were reviewed and approved by the ethical board of the Ministry of Health in Suriname. Written informed consent to participate in this study was provided by the participant or the participants' legal guardian/next of kin.

## Author Contributions

TL, LD, EG, and HY designed the study. TL, IR, LD, HY, and MB recruited the patients and collected the data. TL and FC performed the virus neutralization tests. CG, TL, MK, and BR interpreted the serological data. BJ and KA interpreted the clinical data. IR and TL wrote the draft manuscript. All authors participated in writing the final version of the manuscript.

## Conflict of Interest

The authors declare that the research was conducted in the absence of any commercial or financial relationships that could be construed as a potential conflict of interest.
